# New probes used for IS*1245 *and IS*1311 *restriction fragment length polymorphism of *Mycobacterium avium *subsp. *avium *and *Mycobacterium avium *subsp. *hominissuis *isolates of human and animal origin in Norway

**DOI:** 10.1186/1471-2180-7-14

**Published:** 2007-03-05

**Authors:** Tone Bjordal Johansen, Ingrid Olsen, Merete Rusås Jensen, Ulf R Dahle, Gudmund Holstad, Berit Djønne

**Affiliations:** 1 Department of Animal Health, National Veterinary Institute, PO Box 8156, 0033 Oslo, Norway; 2 Norwegian Institute of Public Health, PO Box 4404 Nydalen, 0403 Oslo, Norway; 3 Norwegian School of Veterinary Science, PO Box 8146, 0033 Oslo, Norway

## Abstract

**Background:**

*Mycobacterium avium *is an environmental mycobacterium that can be divided into the subspecies *avium*, *hominissuis, paratuberculosis *and *silvaticum*. Some *M. avium *subspecies are opportunistic pathogens for animals and humans. They are ubiquitous in nature and can be isolated from natural sources of water, soil, plants and bedding material. Isolates of *M. avium *originating from humans (n = 37), pigs (n = 51) and wild birds (n = 10) in Norway were examined by IS*1245 *and IS*1311 *RFLP using new and specific probes and for the presence of IS*901 *and IS*Mpa1 *by PCR. Analysis and generation of a dendrogram were performed with the software BioNumerics.

**Results:**

IS*1311 *RFLP provided clear results that were easy to interpret, while IS*1245 *RFLP generated more complex patterns with a higher discriminatory power. The combination of the two methods gave additional discrimination between isolates. All avian isolates except one were *M. avium *subsp. *avium *with two copies of IS*1311 *and one copy of IS*1245*, while the isolates of human and porcine origin belonged to *M. avium *subsp.*hominissuis*. The isolates from human patients were distributed randomly among the clusters of porcine isolates. There were few identical isolates. However, one isolate from a human patient was identical to a porcine isolate. Regional differences were detected among the porcine isolates, while there was no clustering of human isolates according to type of clinical symptoms or geographical location of the patient's home addresses.

**Conclusion:**

The results demonstrate that a wide range of *M. avium *subsp.*hominissuis *are present in pigs and humans in Norway, and that some of these isolates are very similar. It remains to be determined whether humans are infected from pigs or if they are infected from common environmental sources.

## Background

*Mycobacterium avium *is an environmental mycobacterium that can be divided into the subspecies *avium*, *hominissuis, paratuberculosis *and *silvaticum *[1,2]. *M. avium *is ubiquitous in nature and can be isolated from natural sources of water, soil, plants and bedding material [3,4].

*M. avium *subsp. *avium *and *hominssuis *are opportunistic pathogens for animals and humans [3,5]. They can cause generalised tuberculosis in poultry and wild birds, while mammals, especially pigs, usually develop localised lesions limited to the lymph nodes of the digestive tract [5]. Previously, lung infections were the most common manifestation of disease due to *M. avium *in humans. Most of these patients had predisposing lung disorders or underlying immunodeficiency. Since the emergence of AIDS, disseminated disease has become more common [3,6]. However, the bacterium can also infect otherwise healthy people, and children can develop subacute lymphadenitis. In Norway, about 100 people get infected with mycobacteria other than those of the *Mycobacterium tuberculosis *complex each year. The majority of these are infected with *M. avium *[7].

The *M. avium *subspecies are a heterogeneous group and strain identification and classification has been based on serotyping and also on molecular methods based on different genomic targets including the presence and distribution of various insertion sequences (IS). Differences between isolates of *M. avium *from birds and the human and porcine isolates have been described. Most avian isolates belong to serotype 1–3 [8], contain IS*901*, and have a distinct pattern by IS*1245 *and IS*1311 *RFLP. They have one copy of IS*1245 *and two copies of IS*1311 *when using the shorter and more specific probes as previously described [9]. Occasionally, humans and pigs may get infected with strains with the classical bird pattern, but generally isolates from humans and pigs do not harbour IS*901*, might harbour IS*Mpa1 *[10] and show another distribution of IS*1311 *and IS*1245 *elements [2,9,11-13]. It was recently proposed to reserve the term *M. avium *subsp. *avium *for strains with the bird pattern, and to call the other strains for *M. avium *subsp. *hominissui*s [2]. Both IS*1245 *and IS*1311 *RFLP have been used to compare isolates from humans and animals in different regions of the world [2,13-15], and their discriminatory power has been judged to be almost equal [12,16-18]. Information about what kind of *M. avium *strains that infect human patients, animals and birds in Norway has not been obtained until now.

IS*1245 *shares an 85% DNA sequence homology with IS*1311 *[16] and the 427 bp IS*1245 *probe used for RFLP by the proposed standardised method [19] share an identity of 82% with IS*1311 *at the DNA level. A problem with the standardised IS*1245 *RFLP method in *M. avium *has been the occurrence of several weak and inconsistent bands, probably due to cross hybridisation [8,16,20]. We previously designed specific probes for IS*1245 *and IS*1311 *that eliminated the possibility of cross hybridisation. Both probes were chosen from the 5' end of each insertion element where there is a 75% homology between the two elements [9].

The aim of this study was to investigate and compare the typing potential of the new probes for IS*1245 *and IS*1311 *RFLP [9], by typing a large number of isolates from different hosts. Furthermore we wanted to examine isolates of *M. avium *subsp. *avium *and *hominssuis *from humans, pigs and wild birds in Norway, in order to determine which type of strains that infect the different hosts. The isolates were compared both by IS*1245 *and IS*1311 *RFLP, and the presence of IS*901 *and IS*Mpa1 *[10]. The study demonstrated that the new probes for IS*1245 *and IS*1311 *RFLP performed well, and that a wide range of *M. avium *subsp. *hominissuis *strains were present in pigs and humans in Norway, and that some of these isolates were very similar.

## Results

**Figure 1:**
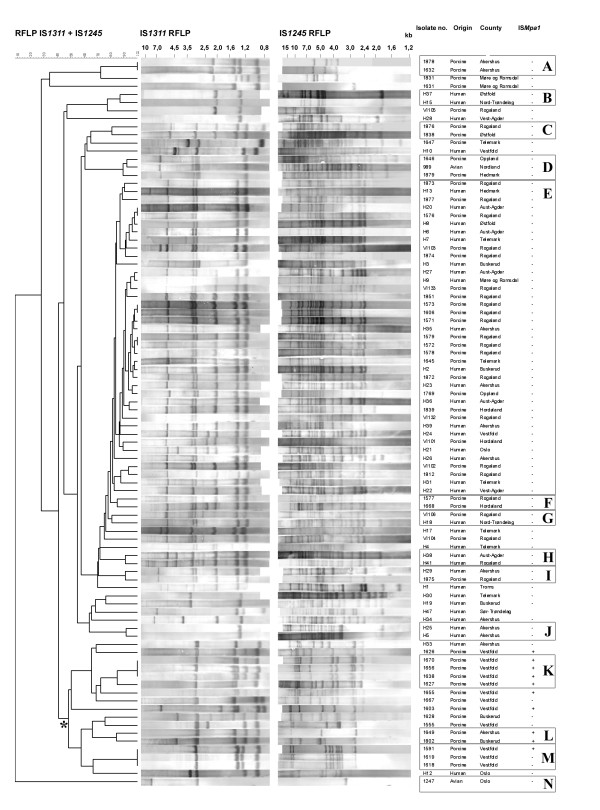
**A dendrogram showing the cluster analysis of the composite dataset of RFLP experiments using IS*1311 *and IS*1245 *as probes**. A total of 98 isolates of *Mycobacterium avium *from Norway are included. The dendrogram was calculated by using the similarity by the average from experiments and the unweighted-pair group method using arithmetic averages clustering method. Correction for internal weights was used. Laboratory numbers, host and county origin of the isolates and the result of the PCR of IS*Mpa1 *are given in columns on the right. The branch that includes the IS*Mpa1 *positive isolates is marked with an asterisk. A total of 14 clusters (defined by 80% similarity) are grouped in rectangular frames and labelled A-N. Cluster N, including nine avian isolates and the reference strain R13, are represented by isolate 1247.

The resulting dendrogram of the cluster analysis is generated from the composite data sets of both the IS*1311 *and IS*1245 *RFLP as described in the BioNumerics manual (Fig 1). Clusters where isolates shared 80% or greater similarity were framed and designated with the capital letters A-N. The *M. avium *isolates demonstrated a high diversity (0.84). The degree of clustering was high (0.78) when the 80% cluster cut off was used. On the other hand, when the cluster cut off was set at 100%, the degree of clustering was very low (0.23).

**Figure 2:**
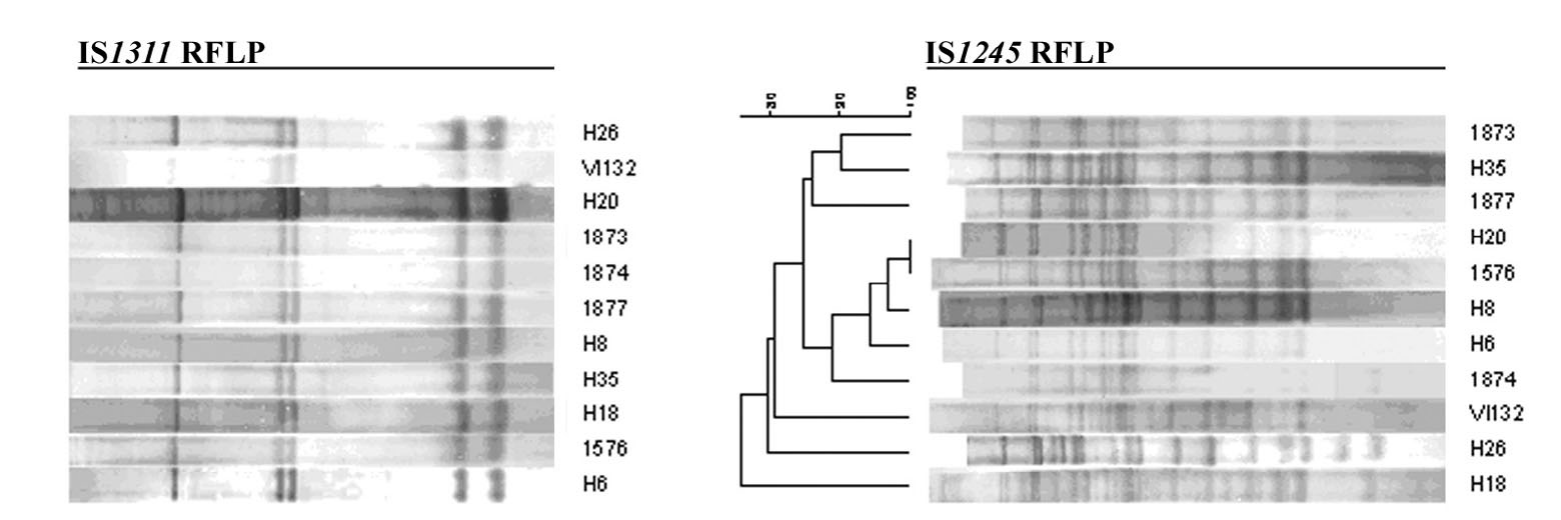
The figure illustrates a set of eleven isolates identical in their banding pattern by IS*1311 *RFLP, which could be further separated by IS*1245 *RFLP. A dendrogram is presented showing the cluster analysis of IS*1245 *RFLP.

RFLP typing with the new and shorter probes reduced the problems of low intensity bands. The *M. avium *isolates included in the current study, carried between one and nine copies of IS*1311*. The RFLP patterns were clear and low intensity bands were only observed for a few isolates. The analysis could be done manually. The current isolates carried between one and 29 copies of IS*1245 *that could be identified by RFLP. Since the isolates carried more copies of IS*1245 *and because this probe produced more low intensity bands than IS*1311*, the IS*1245 *RFLP was more complex and difficult to analyse than the IS*1311 *RFLP. The discriminatory power of the RFLP was, however, higher when the IS*1245 *probe was used. Figure 2 illustrates that a set of eleven isolates with indistinguishable banding pattern by IS*1311 *RFLP, could be differentiated using the IS*1245 *RFLP.

### Bird isolates

Nine bird isolates and the reference strain R13 had identical banding profile with two copies of IS*1311 *and one copy of IS*1245*. This profile is illustrated by only one isolate (#1247) in figure 1, cluster N. The last isolate, #989, which originated from a black grouse, was of the multibanded type with both probes. By use of RFLP, we identified 26 copies of IS*1245*, in addition to 6 copies of IS*1311*. The RFLP patterns of this isolate clustered with two porcine isolates, #1646 and #1879 (Fig. 1, cluster D). The three isolates were identical by IS*1311 *RFLP but slight differences were observed when IS*1245 *RFLP was used. The cluster analysis from the composite data set showed 92% similarity between isolate #989 and isolate #1646 and 89% similarity between #989 and #1879 (Fig. 1). All bird isolates except #989 harboured IS*901*, and none harboured the IS*Mpa1 *element.

### Porcine and human isolates

All isolates from swine and humans were *M. avium *subsp. *hominissuis*; they lacked IS*901*, and none of them showed the bird pattern on IS*1245 *and IS*1311 *RFLP. The majority of the isolates were multibanded; however, some had only one or two copies of IS*1311 *that could be identified by RFLP. One copy of IS*1245 *and two copies of IS*1311 *were observed for the porcine isolate #1631, but the pattern was different from that observed in isolates from birds.

**Figure 3:**
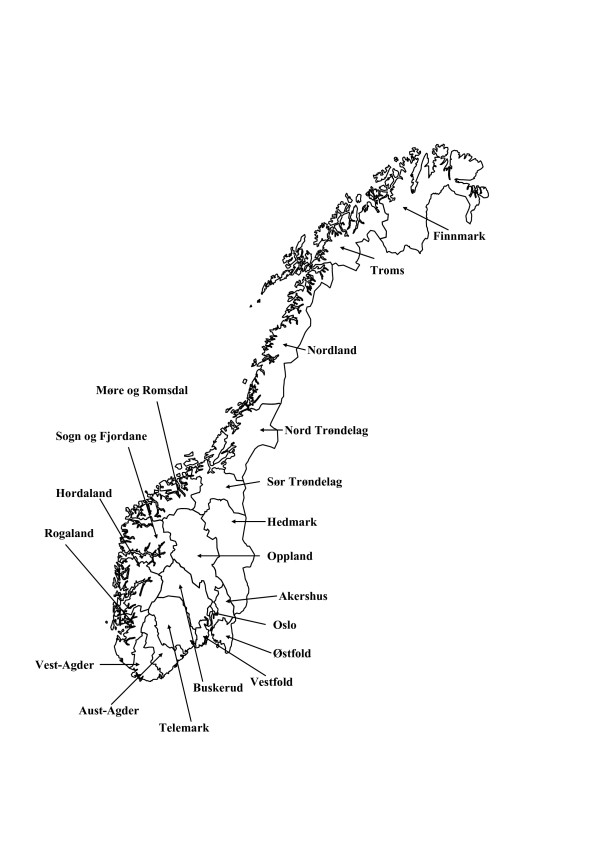
A map of Norway showing the different counties.

Several different RFLP patterns were detected, and a few isolates with identical patterns were found. The isolates of porcine and human origin were grouped into thirteen clusters, named A-M, (Fig. 1). The largest cluster, cluster E contained 39 isolates. Among them, 21 were of porcine origin, and 17 (81%) of these originated from the same county; Rogaland (Fig. 3). Among the remaining 30 porcine isolates that were not included in cluster E, only six (20%) originated from Rogaland. Two sets of the porcine isolates in cluster E were identical by both RFLPs, #1579 and #1572, and #1606 and #1573. These isolates originated from Rogaland, but not from the same farms. The 18 isolates of human origin that were assigned to cluster E were distributed between the porcine isolates, and the patients lived in different counties in Norway. One of these (H20) was identical to one isolate of porcine origin (#1576) by IS*1311 *and IS*1245 *RFLP. The pig originated from Rogaland and the person was a resident of the neighbouring county Aust-Agder. All isolates in cluster E were negative for IS*Mpa1*.

The remaining 12 clusters, A-D and F-M, included 2–4 isolates. Two clusters included three porcine isolates (cluster K and M) that carried 100% identical IS*1311 *and IS*1245 *RFLP patterns. Also, cluster A included two porcine isolates that were identical by both RFLP methods. The identical isolates originated from the same counties, but not from the same farms.

Among the porcine isolates, ten were positive for IS*Mpa1*, none of the isolates of human or avian origin harboured this element. All IS*Mpa1 *positive isolates were grouped separately from most of the other isolates. The branch that includes these ten isolates is labelled with an asterisk (*) in figure 1. The porcine isolates in this branch all came from the same part of Norway, mainly from Vestfold, but also from the two neighbouring counties Akershus and Buskerud (Fig. 3).

Four farms were represented by more than one porcine isolate. Of these, #1591 and #1603 were isolated from one farm, #1618, #1626 and #1627 from a second farm, #1619 and #1670 from a third farm and #1649 and #1878 from a fourth farm. None of them were identical to other isolates from the same farm, and the similarity between them was quite low, ranging from 66% (#1626 and #1627) to 28% (#1649 and #1878). Interestingly, among these nine isolates, four carried RFLP patterns that were 100% identical to at least one isolate from another farm in the same county.

The isolates of human origin were distributed randomly among the isolates from pigs. There was no clustering of isolates according to type of clinical symptoms or to geographical location of the patients' home addresses (Table 1 and 2).

## Discussion

The new short probes performed well for typing *M. avium *isolates. Based on these probes, it was demonstrated for the first time in Norway that isolates from birds were mainly *M. avium *subsp. *avium *and that isolates from humans and pigs were *M. avium *subsp. *hominissui*s. A geographical distribution of isolates of porcine origin was found, and similar isolates from humans and pigs were common.

The two probes differed in their typing potential and could have different areas of application. The IS*1311 *RFLP gave fewer bands than the IS*1245 *RFLP on the majority of the human and porcine isolates. This made the interpretation of the analysis easier, but the discriminatory power was weaker than for the IS*1245 *RFLP. In other studies the discriminatory power of the two RFLP methods have been almost equal [12,16-18]. The IS*1311 *RFLP patterns can be analysed manually, if expensive software is not available. Comparison of IS*1311 *RFLP and IS*1245 *RFLP showed that IS*1311 *RFLP can be a good choice for several typing purposes. One can improve the separation of *M. avium *isolates by combining the two methods. For example, the IS*1311 *RFLP could be useful when comparing isolates of *M. avium *from different countries or parts of the world. The interpretation of results from this method is fairly easy to calibrate, since it generates fewer bands and because low intensity bands are uncommon. In an international setting the IS*1311 *RFLP may be applied to detect major differences between isolates within *M. avium*. The IS*1311 *RFLP is also considered to be well suited for a primary screening when isolate comparison is needed. When the discrimination of isolates has to be of a higher level, such as in outbreak investigations, inquiries of possible laboratory contamination and in contact tracing, the combination of the two RFLP methods appears to be the ideal.

Nine of ten avian isolates in our material belonged to the well described bird type of *M. avium *subsp.*avium*. The isolate #989 that originated from a black grouse was negative for IS*901 *and had a multibanded profile with IS*1311 *and IS*1245 *RFLP. The finding of a *M. avium *subsp. *hominissuis *isolate from birds seems to be an exception, but has been described earlier [11,21,22]. *M. avium *subsp. *avium *is the cause of avian tuberculosis and is known to be a contagious disease among birds [5], while *M. avium *subsp. *hominissuis *probably behaves as an opportunistic environmental pathogens in birds like it does in mammals.

The isolates of human and porcine origin in our material were all *M. avium *subsp. *hominissuis*. Most of these isolates had multibanded RFLP profiles on both IS*1311 *and IS*1245 *and none harboured IS*901*. A few isolates showed a low copy number, especially with IS*1311 *RFLP, and one isolate (#1631) had only one copy of IS*1245*. However, these isolates were all easily differentiated from the bird type. *M. avium *subsp.*avium *has been detected in human patients [21,22], pigs and other animals [12,21,22], however this seems to be rare. Many *M. avium *subsp. *hominissuis *isolates of human and porcine origin were clustered together when the cut off was set at 80%, and one porcine and one human isolate were identical (H20 and #1576).

The isolates from human patients did not show any significant clustering with respect to clinical symptoms, age or immune status. This was in accordance with earlier studies; since differences between isolates from children and adults and isolates from HIV infected and not HIV infected patients could not be detected [21,22].

The porcine isolates that were grouped into the same clusters with 80% or greater similarity generally originated from the same geographical areas. A total of 17 of 23 isolates from Rogaland (73.9%) belonged to the largest cluster, cluster E. In this county, there was a special focus on environmental mycobacteria in the period from 1986 to 2000. An unusual number of children at the schools of Karmøy, Rogaland showed hypersensitivity reactions when challenged with the tuberculin skin test without any signs of infection with bacteria in the *M. tuberculosis *complex. The reactions were suspected to be due to a sensitisation to environmental mycobacteria, especially *M. avium*. At the same time personnel at a slaughter house in Rogaland showed increased reactions to the skin test following a period of an unusually high occurrence of mycobacteriosis in swine at the same slaughterhouse [23]. The number of swine with tuberculous lesions in Norway varies between areas and from year to year. There have been few investigations to find the prevalence of these lesions, but one investigation at the meat inspection in Haugesund (also in Rogaland county) performed between 1986 and 1990 found tuberculous lesions in about 0.5–1.5% of the swine slaughtered [24].

Our data suggest that birds are not a natural source of infection with *M. avium *for humans or swine in Norway, and our findings are similar to those reported in other studies [2,12,13,15]. However, in a Swedish study, about half of the isolates from pigs were of the bird type, indicating that birds might be the cause of infection for pigs [21]. In the present study, humans and swine might have infected each other, although we consider it more likely that the two species are infected from similar environmental sources. This was strengthened by the observation that among animals from the same farms, different *M. avium *subsp.*hominissuis *isolates were found. The geographical clustering of the porcine isolates in our material also suggest that bacteria in the same area are more closely related to each other than to bacteria in another areas, and that pigs on different farms may have been infected from the same environmental source. *M. avium *has been isolated from different kinds of vegetables [25,26], and a food isolate was identical to an isolate from a human patient when compared by a PCR based typing method [27]. Other potential environmental sources of mycobacteria for both humans and swine are soil and water. *M. avium *has been isolated from potted plants [26], and from peat [22,28]. Identical isolates of *M. avium *have been found in samples from peat and human patients [22] and in samples from peat and pigs [28]. It appears that soil and water may represent the *M. avium *reservoir for mammals, including humans, in Norway as in other countries [29,30].

## Conclusion

Our study demonstrated that the new probes for IS*1311 *and IS*1245 *RFLP performed well for typing the isolates, and that *M. avium *subsp. *avium *were detected only among birds in Norway. In pigs and humans, however, a wide range of *M. avium *subsp.*hominissuis *are present and some of these isolates are very similar. It remains to be determined whether humans are infected from pigs or if they are infected from common environmental sources.

## Methods

### Bacterial isolates

Ninety-eight isolates of *M. avium *were examined. The isolates were collected from humans (n = 37), pigs from 46 farms (n = 51) and birds (n = 10). All isolates and their origin are shown in tables 1 and 2. The human isolates were received from Rikshospitalet University Hospital and from The Norwegian Institute of Public Health. The isolates from the latter institution originated from various hospitals in Norway. The isolates originated from immunocompromised patients with and without HIV and from non-immunocompromised patients, including children with lymphadenitis and adults with pulmonal disease (Table 2). The porcine isolates came from slaughter house material from different parts of Norway. Eight avian isolates came from wild birds of prey and two isolates from other wild birds. They originated from diseased birds from different parts of the country (Table 1). Avian tuberculosis in poultry has not been diagnosed in Norway during the last 20 years (M. Kalhusdal, personal communication), and no such isolates were therefore available. All samples of animal origin were isolated at The National Veterinary Institute of Norway, the bird isolates in the period 1989 to 2004 and the porcine isolates between 1994 and 2002. Primary isolation was performed as described by Valheim et al [31]. Briefly, approximately two grams of tissue from lymph nodes or internal organs were homogenized and decontaminated with 5% oxalic acid, centrifuged, redissolved in saline water, inoculated on Petragani medium, Stonebrinks medium and Middlebrook 7H10 (Difco Laboratories, Detroit, MI) and incubated at 37°C for up to two months. Ziehl-Neelsen positive isolates were confirmed as *M. avium *by Accu Probe (GenProbe Inc., San Diego, CA) and stored in Middlebrook 7H9 supplemented with 10% (v/v) OADC (Difco) and 0.2% (v/v) glycerine (Merck KGaA, Darmstadt, Germany) at -70°C. For examination, the isolates were cultured on Løwenstein Jensen medium (Difco) or on Middlebrook 7H10 supplemented with 10% (v/v) OADC Enrichment (Difco) at 37°C until sufficient growth.

### PCR

All isolates were examined for the presence of IS*Mpa1 *and IS*901 *by PCR using 1 U AmpliTaq^® ^DNA polymerase (Applied Biosystems, Foster City, CA). Primers P2 and P3 were used for amplification of IS*Mpa1 *[10], and primers 901a and 901c for the amplification of IS*901 *[32]. The amplification of IS*Mpa1 *was performed by an initial denaturation step at 94°C for 3 min followed by 30 cycles of denaturation at 94°C for 30 s, annealing at 60°C for 30 s and extension at 72°C for 1 min. The IS*901 *PCR was performed with a different annealing temperature, 55°C, and with 35 cycles instead of 30. Otherwise, the conditions were identical for both PCRs. The PCR products were analysed on a 2% (w/v) agarose gel.

### Restriction fragment length polymorphism (RFLP)

The IS*1245 *and IS*1311 *RFLP analyses were performed as previously described [9,10,19]. The bird strain R13 was used as a positive control and run on each gel [19]. The probes used for IS*1245 *and IS*1311 *RFLP were described earlier [9].

The resulting RFLP patterns were analysed by visual inspection and by using the BioNumerics software (version 3.5; Applied Maths, Kortrijk, Belgium). Normalisation of the fingerprints was done using the molecular weight standard 1 Kb DNA Ladder (Invitrogen™, Carlsbad, Calif.), which wasrun in three lanes per gel. The software was used to calculate Dice coefficients of similarity, to cluster the isolates and to generate dendrograms by the unweighted-pair group method using average linkages. This was done for each RFLP (IS*1245 *and IS*1311*) separately. The most appropriate settings for optimization and tolerance, as determined by the software, were calculated. For IS*1311 *RFLP the calculated optimization and tolerance settings were 0.40% and 0.87%, respectively, while for the IS*1245 *RFLP the settings were 0.30% and 0.21%, respectively. These settings were considered too strict by visual analysis. The control strain (R 13) clustered at less than 100% similarity, and other isolates that seemed identical by manual analysis, did not cluster with 100% similarity. Instead, both the optimization and tolerance settings of 1.5% were chosen for IS*1311 *RFLP and of 0.7% for the IS*1245 *RFLP. With these settings, R13 clustered at 100% similarity. A final dendrogram of the composite dataset of the two RFLP experiments was calculated using the similarity by the average from experiments, and the UPGMA clustering method. Correction for internal weights was used. The cluster cut off was set at 80%.

The degree of diversity was calculated by dividing the number of different RFLP patterns by the number of isolates analysed. The degree of clustering was calculated by dividing the total number of isolates that clustered by the number of isolates analysed [33,34]. PCR results for IS*901 *and IS*Mpa1 *are not included in the cluster analysis.

## Authors' contributions

TBJ contributed to conception and design, laboratory experiments, analysed data and drafted the manuscript. IO, BD and GH contributed to conception and design, data analysis and the writing of the manuscript. MRJ contributed to the laboratory experiments and writing of the manuscript and UD supplied human isolates, contributed to establish the method, data analysis and in writing of the manuscript. All authors read and approved the final manuscript.

## Figures and Tables

**Table 1:** The table illustrates all isolates of *Mycobacterium avium *examined, their host and county of origin.

County of origin	Avian isolates	Porcine isolates	Human isolates
Troms	1641, 1882		H1
Nordland	989		
Nord Trøndelag			H15, H18
Sør Trøndelag			H47
Møre og Romsdal		1631, 1831	H9
Hordaland		1668, 1839, VI101	
Rogaland		1571, 1572, 1573, 1576, 1577, 1578, 1579, 1606, 1812, 1851, 1872, 1873, 1874, 1875, 1876, 1877, VI102, VI103, VI104, VI105, VI106, VI132, VI133	H41
Vest Agder			H22, H28
Aust Agder			H6, H20, H27, H36, H38
Telemark		1645, 1647	H4, H7, H17, H30, H31
Vestfold	1553, 1871	1555, 1591, 1603, 1618, 1619, 1626, 1627, 1638, 1655, 1656, 1667, 1670	H10, H24
Østfold		1838	H8, H37
Oslo	1247, 1867,		H12, H21
Akershus	1688	1632, 1649, 1878	H5, H23, H25, H26, H29, H33, H34, H35, H39
Buskerud		1628, 1802,	H2, H3, H19
Oppland	1794	1646, 1769	
Hedmark	1608	1879	H13

**Table 2:** The table illustrates the clinical site from which the human isolates of *Mycobacterium avium *were isolated.

Isolation site	Isolate number
Expectorate or lungs	H1, H5, H6, H9, H10, H12, H15, H18, H36^1^, H37^1^, H38^1^, H39^1^, H47
Head lymph nodes	H3, H4, H7, H8, H13, H19, H20, H21, H22, H23, H24, H25, H26, H27, H28^1^, H29
Abscess	H17
Blood culture	H2, H41^2^
Unknown site	H30^1^, H31^1,2^, H33^1,2^, H34^1,2^, H35^1,2^

Isolates from immunocompromised patients are marked with ^1 ^and HIV positive with ^2^. Immune status for patients is unknown for isolate H1-H19 and H47.
